# Differentiation of granulomatous nodules with lobulation and spiculation signs from solid lung adenocarcinomas using a CT deep learning model

**DOI:** 10.1186/s12885-024-12611-0

**Published:** 2024-07-22

**Authors:** Yanhua Wen, Wensheng Wu, Yuling Liufu, Xiaohuan Pan, Yingying Zhang, Shouliang Qi, Yubao Guan

**Affiliations:** 1 Department of Medical Imaging, the Fifth Affiliated Hospital of Guangzhou Medical University, 621 Gangwan Road, Guangzhou, 510700 Guangdong China; 2 Department of Radiology, The First Affiliated Hospital of Guangzhou Medical University, Guangzhou, Guangdong China; 3 Key Laboratory of Intelligent Computing in Medical Image, College of Medicine and Biological Information Engineering, Northeastern University, Shenyang, Liaoning China

**Keywords:** Deep learning, Granulomatous nodules, Lung adenocarcinomas, Computer tomography, Artificial Intelligence

## Abstract

**Background:**

The diagnosis of solitary pulmonary nodules has always been a difficult and important point in clinical research, especially granulomatous nodules (GNs) with lobulation and spiculation signs, which are easily misdiagnosed as malignant tumors. Therefore, in this study, we utilised a CT deep learning (DL) model to distinguish GNs with lobulation and spiculation signs from solid lung adenocarcinomas (LADCs), to improve the diagnostic accuracy of preoperative diagnosis.

**Methods:**

420 patients with pathologically confirmed GNs and LADCs from three medical institutions were retrospectively enrolled. The regions of interest in non-enhanced CT (NECT) and venous contrast-enhanced CT (VECT) were identified and labeled, and self-supervised labels were constructed. Cases from institution 1 were randomly divided into a training set (TS) and an internal validation set (IVS), and cases from institutions 2 and 3 were treated as an external validation set (EVS). Training and validation were performed using self-supervised transfer learning, and the results were compared with the radiologists’ diagnoses.

**Results:**

The DL model achieved good performance in distinguishing GNs and LADCs, with area under curve (AUC) values of 0.917, 0.876, and 0.896 in the IVS and 0.889, 0.879, and 0.881 in the EVS for NECT, VECT, and non-enhanced with venous contrast-enhanced CT (NEVECT) images, respectively. The AUCs of radiologists 1, 2, 3, and 4 were, respectively, 0.739, 0.783, 0.883, and 0.901 in the (IVS) and 0.760, 0.760, 0.841, and 0.844 in the EVS.

**Conclusions:**

A CT DL model showed great value for preoperative differentiation of GNs with lobulation and spiculation signs from solid LADCs, and its predictive performance was higher than that of radiologists.

**Supplementary Information:**

The online version contains supplementary material available at 10.1186/s12885-024-12611-0.

## Introduction

Solitary pulmonary nodules (SPNs) are round lesions < 3 cm in diameter that are surrounded by normal lung tissue without atelectasis, hilar enlargement, or pleural effusion [1]. With the increasing use of low-dose CT screening and improved awareness of health examinations, the SPNs detection rate has increased significantly [2]. However, despite the importance of distinguishing benign and malignant SPNs, some SPNs are difficult to assess qualitatively on the basis of radiologist assessments alone [3, 4]. Although most solid SPNs with lobulation and spiculation signs are lung adenocarcinomas (LADCs), some have been pathologically confirmed as granulomatous nodules (GNs) postoperatively [5, 6]. For the lobulation sign, up to25% were confirmed to be benign lesions; while 88–94% of the spiculation sign indicated malignancy, but a few, such as tuberculoma, were confirmed to be benign [6]. PET/CT can evaluate the benign and malignant properties of SPNs on the basis of the presence or degree of fluorodeoxyglucose uptake. However, PET/CT is expensive, not sensitive to pulmonary nodules with a diameter of 8–10 mm, and may show false-negative or false-positive results for SPNs diagnoses [7, 8]. Although MRI can also distinguish benign and malignant lesions, it shows insensitivity for small lesions, low resolution of the lung structure, and the propensity to be easily disturbed by motion artifacts [9, 10]. Consequently, distinguishing GNs with lobulation and spiculation signs from solid LADCs has remained difficult in clinical practice.

In traditional imaging diagnosis based on human vision, many minor and important signs are easily overlooked, and the assessments are often subjective and empirical. Moreover, overlaps in the imaging features of different lesions can restrict even experienced radiologists from providing definitive diagnoses, and human observers cannot easily evaluate and predict deep-level information inside the tumor. These limitations highlight the need for informative, standardized, reproducible, and highly efficient methods to assist and enhance imaging diagnoses.

Deep learning (DL), an important branch of machine learning, involves learning representations from data with an emphasis on learning from connected layers corresponding to increasingly meaningful representations [11, 12]. Compared with traditional machine learning techniques, DL is capable of recognizing lesions in images with high accuracy and automatically extracting lesion features for end-to-end computation, which effectively avoids manual segmentation of lesions and complex non-automatic feature extraction processes [11–13]. In this study, a CT DL model based on self-supervised transfer learning was constructed, and its predictive performance was compared with that of radiologists to explore its predictive value for distinguishing GNs with lobulation and spiculation signs from solid LADCs.

## Methods

### Study population

The inclusion criteria were as follows: (1) pathologically confirmed GNs or LADCs; (2) plain CT and contrast-enhanced examinations performed within 2 weeks before surgery and images reconstructed with slice thickness ≤ 2 mm; (3) lesions appearing as solid SPNs without calcification and fat inside, lobulation and spiculation signs on the margin, and a diameter of 8–30 mm; (4) availability of complete clinical and imaging data.

The exclusion criteria were as follows: (1) pathologically confirmed other types of tumors; (2) CT images with severe artifacts or suboptimal image quality or reconstructed with thickness values > 2 mm; (3) lesion diameter not in the range of 8–30 mm; (4) lesions appearing as subsolid nodules on CT, containing fat or calcification within the lesion, or showing a regular margin ; (5) incomplete clinical or imaging data.

**Fig. 1:**
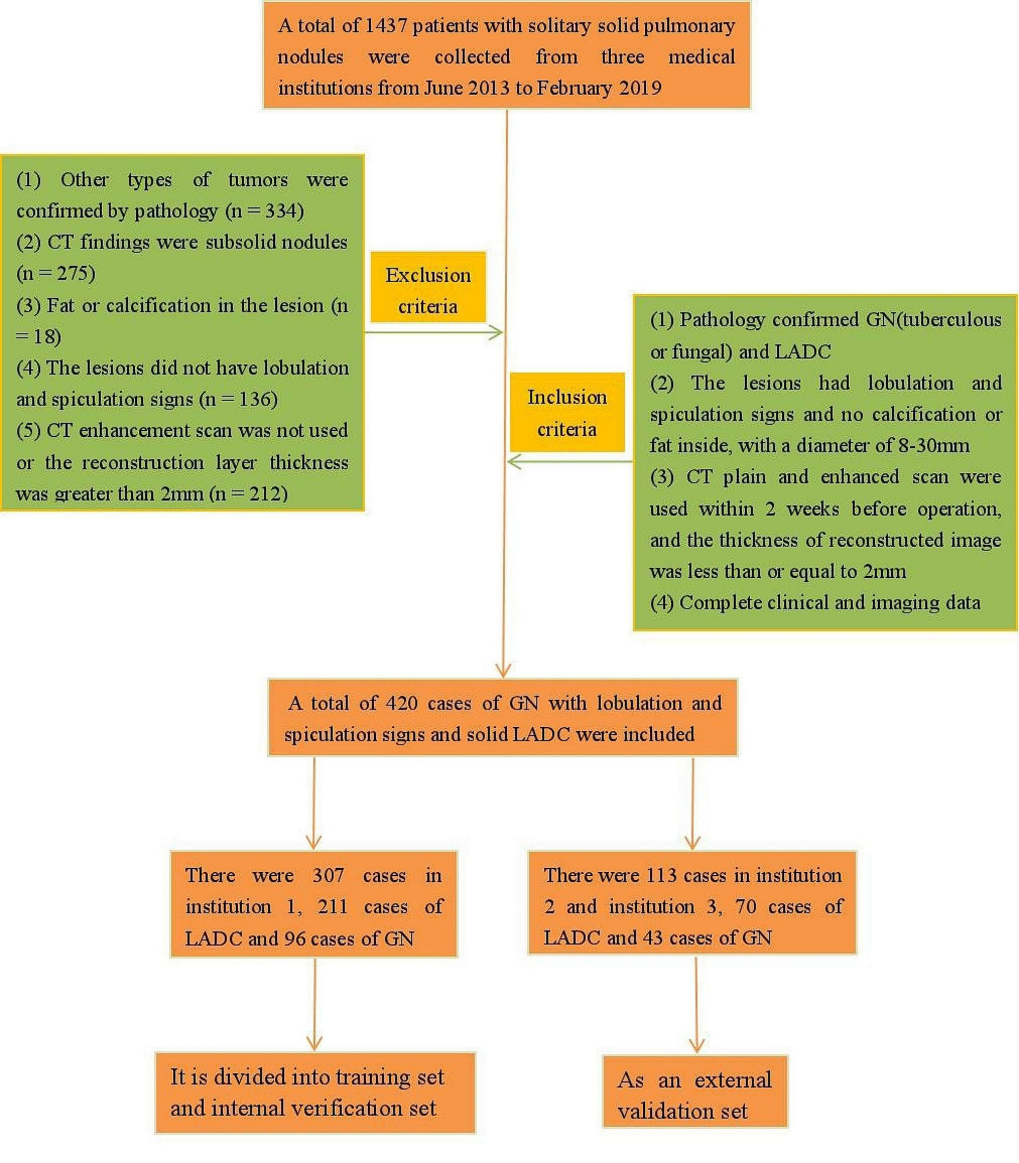
The case screening process of this study

Based on the above inclusion and exclusion criteria, we retrospectively recruited a total of 420 patients with a pathologically confirmed diagnosis of GNs or LADCs by surgical resection or puncture biopsy between June 2013 and February 2019 from three medical institutions. There were 307 cases in Institution 1 (211 LADCs and 96 GNs) and 113 cases in Institutions 2 and 3 (70 LADCs and 43 GNs). In this study, we randomly divided the cases from Institution 1 into training set (TS) and internal validation set (IVS), and used the cases from Institutions 2 and 3 as external validation set (EVS). The case screening process is depicted in Fig. 1.

### CT image acquisition

Images were acquired at the three hospitals with Siemens Definition AS + 128-slice and 64-slice CT, Canon 640-slice CT (Aquilion ONE Vision), and Ge Optima CT660 64-row 128-slice CT scanners. All patients underwent plain and enhanced CT; the scan range was from the tip of both lungs to the costophrenic angle bilaterally. Scanning parameters were as follows: tube voltage, 120 kV; automated tube current; acquisition matrix, 512 × 512; and field of view, 500 mm × 500 mm. The mediastinal and lung windows were reconstructed using standard algorithms.

### Image preprocessing and lesion extraction

**Fig. 2:**
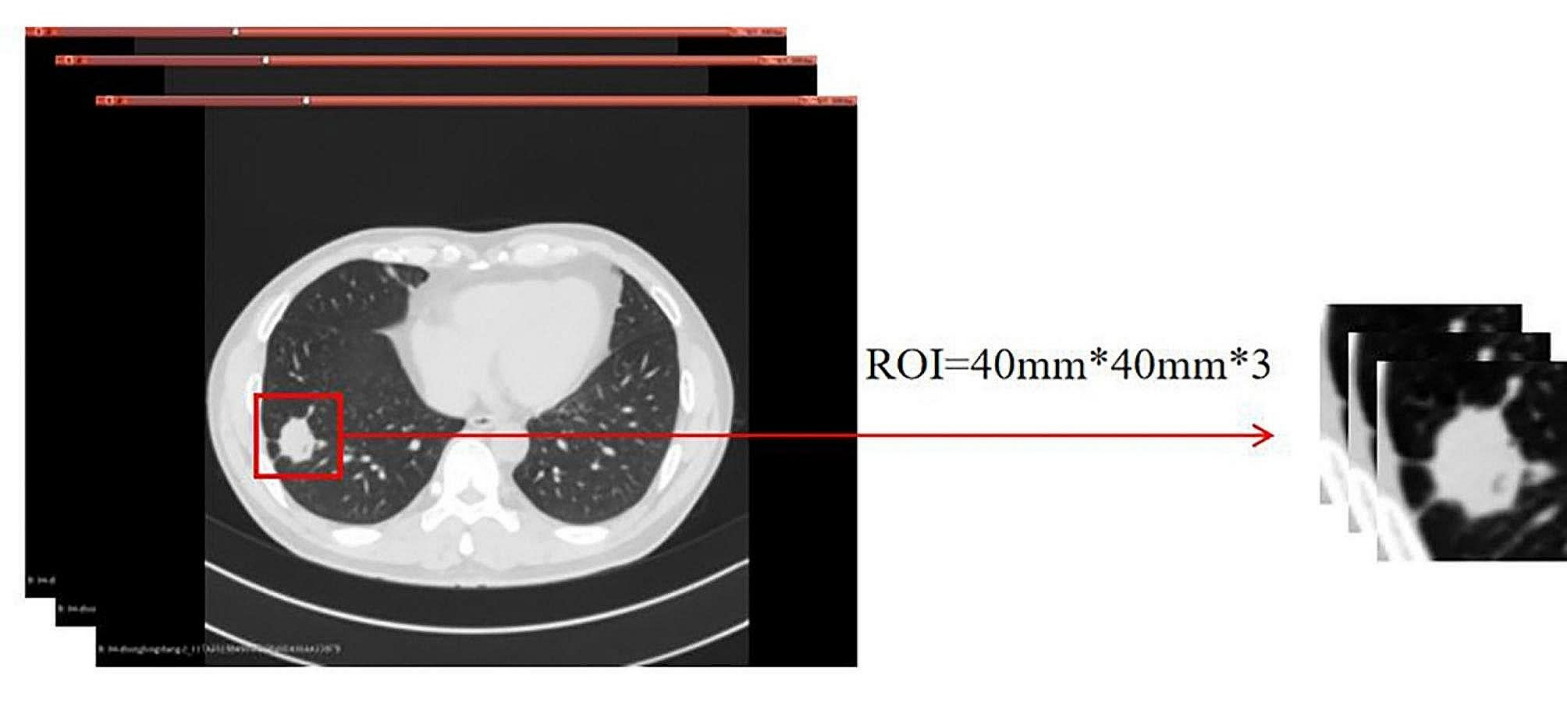
Region of interest extraction process. The 3D slicer software was applied to obtain the region of interest (ROI): first, the coordinates of the centre of the lesion were used as the datum, then coordinate points 1 mm above and 1 mm below the centre coordinates were determined, and finally a square ROI of 40 mm in diameter was obtained with each of these three points as the centre. Since the lesions we included were pulmonary nodules with a diameter of < 30 mm, the ROIs obtained for each lesion were three slices containing the entire cross-section of the lesion

Image preprocessing: (1) the image was resampled using a linear interpolation algorithm, with the sampling layer thickness interval set to 1 mm (although the interpolation algorithm is somewhat detrimental to the detection of edge features, it ensures isotropy of the image voxels and has higher spatial positional precision [14] ); (2) the window width and window position were adjusted to 1400 HU and − 500 HU for all images. The 3D slicer software was applied to obtain the region of interest (ROI): first, the coordinates of the centre of the lesion were used as the datum, then coordinate points 1 mm above and 1 mm below the centre coordinates were determined, and finally a square ROI of 40 mm in diameter was obtained with each of these three points as the centre. Simultaneously, the GNs and LADCs were identified as benign and malignant, respectively. The ROI extraction process is shown in Fig. 2.

### Definition of labels

Our study adopts a self-supervised learning approach [15], and its main process is divided into two stages: (1) self-supervised pretext task training: this stage entails the design of a pretext task, and a pseudo label for the pretext task is automatically generated for the unlabelled data based on certain attributes of the data (e.g., image panning, flipping, rotating, etc.), and after completing the training with the pseudo label, a network model that is capable of capturing the visual features of the image is obtained; (2)supervised downstream task training: after self-supervised pretext task training finished, the learned parameters serve as a pre-trained model and are transferred to other downstream computer vision tasks by fine-tuning.

It has been shown that the method of using rotation to generate pseudo labels in self-supervised learning can achieve good results in visual, audio, text and other tasks [16]. In this study, the obtained ROI patches were transformed to generate pseudo labels by doing four angles (each patch was rotated counterclockwise by 0°, 90°, 180°, and 270°, respectively), which was done in order to enable the CNN to learn to recognise and detect the local features of the lesions [16]. However, this approach somewhat ignores the overall features of the lesion. We designated the image rotation angle as the pseudo label and the lesion benignity and malignancy as the original label. In addition, the geometric transformation (i.e., rotation) of the image carried out by this step achieves data augmentation and avoids the problem of unbalanced samples in this study.

### Model construction

**Fig. 3:**
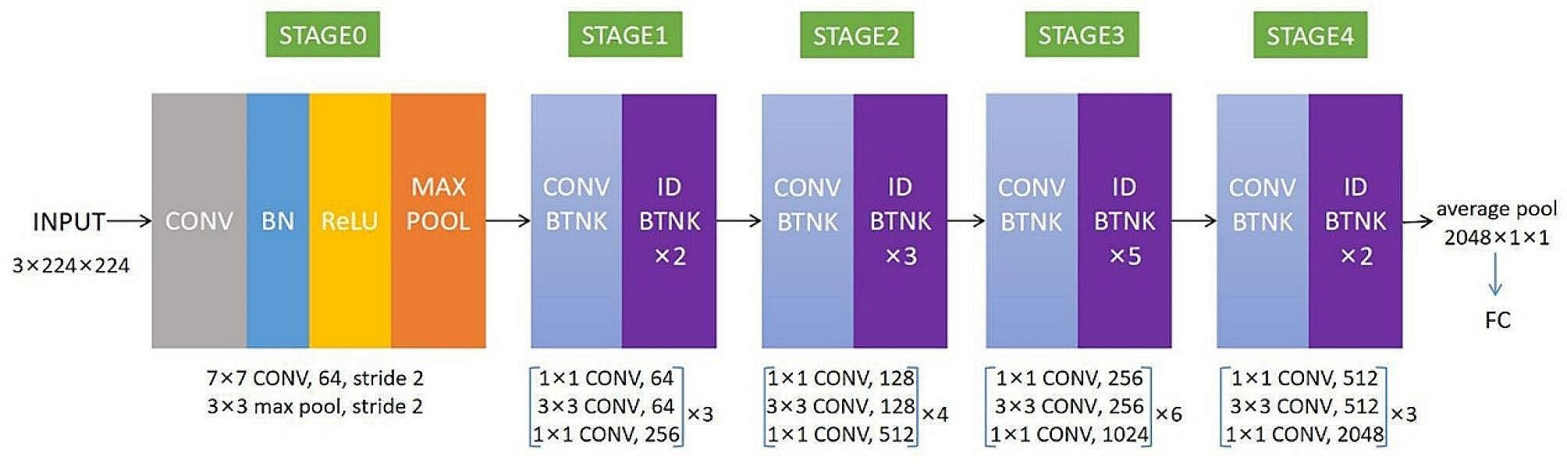
The structure of ResNet50 and parameters during training. ResNet50 includes 49 convolution layers and one full connection layer from input to output, which can be divided into five stages. The structure of the first stage is relatively simple and can be regarded as the pretreatment of input. The last four stages are composed of bottlenecks, and their structures are relatively similar. CONV represents the convolution layer, which is used to extract features; Maxpool indicates the maximum pooling operation, which can avoid overfitting; Relu refers to the activation function, which can accept the signal output from the previous unit and convert it into a form that can be received by the next unit; BN refers to batch normalization processing, which can cut the image data to a specified size; BTNK in stages 1–4 represents the bottleneck structure, and each BTNK contains three convolution layers; FC layer represents the fully connected layer with the functions of combining features and classifying discriminations

**Fig. 4:**
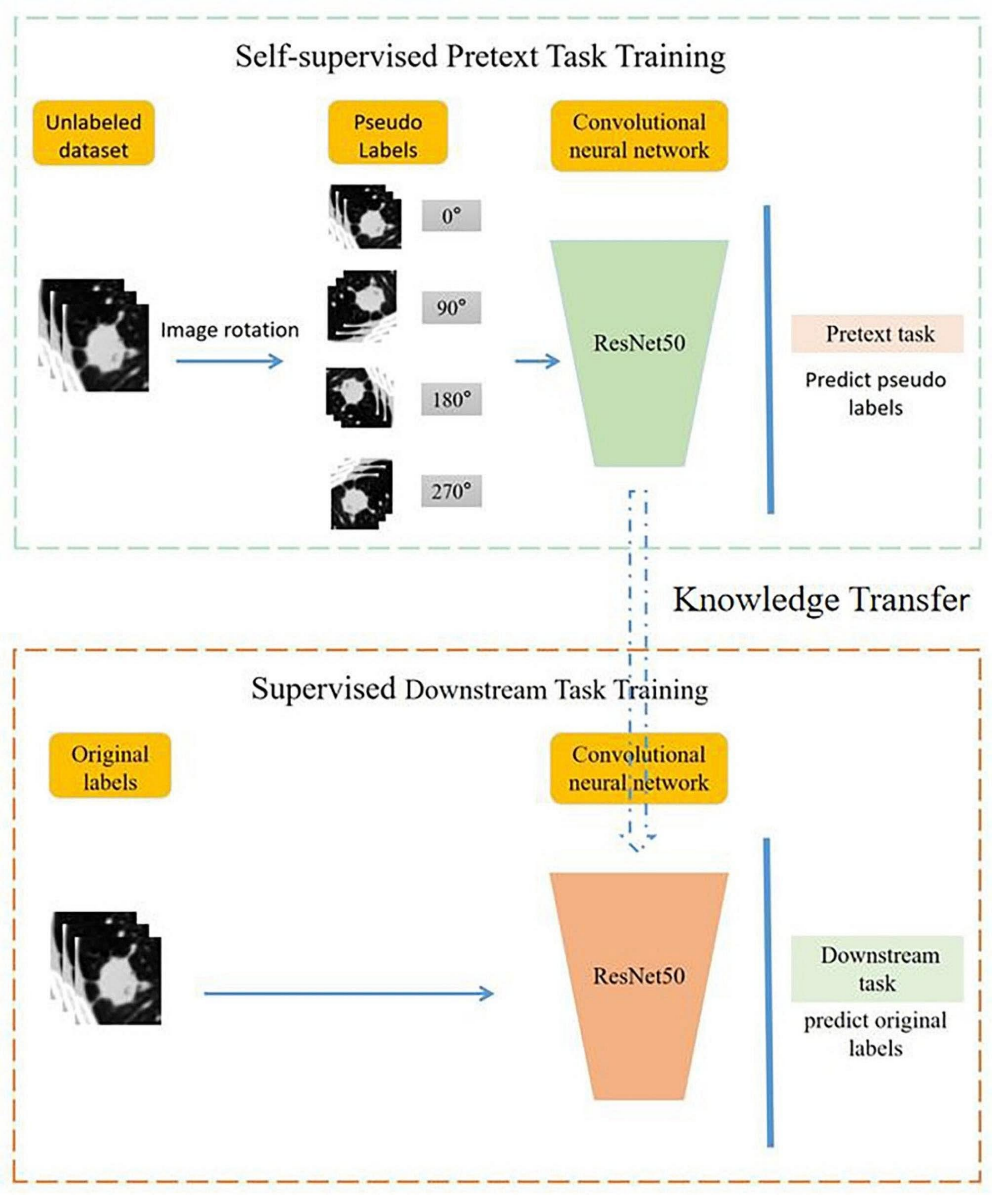
The process of building deep learning models. The process includes two stages, (1) self-supervised pretext task training: the pseudo labels were entered into ResNet50 for DL and predicted. (2) supervised downstream task training: after self-supervised pretext task training finished, the learned parameters serve as a pre-trained model and are transferred to downstream task by fine-tuning (prediction of benign and malignant lesions). The parameters of the training layers are shown in Fig. 3, only the FC layer parameters are different between the two stages, self-supervised pretext task training is (2048,8) and supervised downstream task training fine-tuning for (2048,2)

ResNet50 is a residual network composed of many residual units connected in series. It solves the problems of gradient vanishing and gradient explosion that occur as the network deepens, allowing the network to deepen without degrading performance. Therefore, we choose ResNet50 network to construct the model [17].The residual unit’s structure is shown in Supplementary Fig. 1. The structure of ResNet50 is shown in Fig. 3. Based on the ROI obtained above, DL models of non-enhanced CT (NECT), venous contrast-enhanced CT (VECT), and non-enhanced with venous contrast-enhanced CT (NEVECT) were established, and the IVS was used for internal verification with five cross-validation. Thus, all cases in institution 1 participated in the training and internal verification in this study. Finally, the model was validated using the EVS. The establishment process of the DL model is shown in Fig. 4.

### Radiologist lesion assessment

Two junior radiologists with 3–5 years of work experience (radiologists 1 and 2) and two senior radiologists with 5–10 years of work experience (radiologists 3 and 4) independently evaluated the benign and malignant pulmonary nodules without foreknowledge of the pathology. When the diagnostic results were different, the radiologists discussed and reached a consensus, which was regarded as the Radiologist consensus results (RCR). The four radiologists also measured the lesion diameter, which was considered as the longest diameter measured in the horizontal axis position, and the average value of the four radiologists’ measured values was considered as the final lesion diameter.

### Statistical analysis

SPSS 24.0 (IBM) and Medcalc (version 19.1.2.0) software were used for statistical analysis. The measurement data were expressed as mean ± standard deviation, and the counting data were expressed by frequency. T-tests of two independent samples were performed for comparison of measurement data, and the chi-square test was used for comparison of counting data. When *p* < 0.05, the difference was considered to be statistically significant. The Kappa test was used to test the consistency of the diagnostic results obtained by radiologists. The larger the kappa value, the better the consistency.

Area under curve (AUC) of receiver operating characteristic curve, 95% confidence interval(CI), sensitivity, and specificity were obtained to evaluate the diagnostic efficacy of DL models and radiologists. The Delong test was used to compare differences between different DL models and the diagnostic efficacy of radiologists. When *p* < 0.05, the differences were considered to be statistically significant.

## Results

### General clinical data

**Fig. 5:**
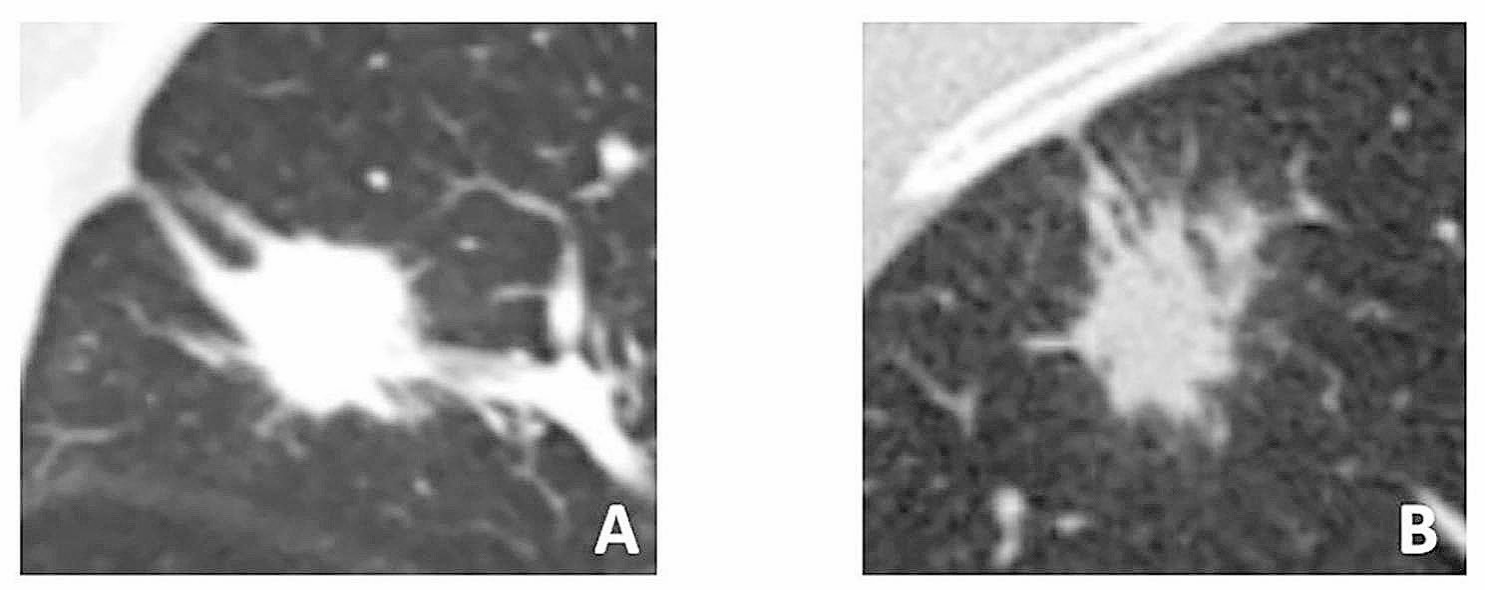
Examples of malignant and benign samples from this study. **A**: The margins of the lesion can be seen with lobulation and spiculation signs, and the lesion was confirmed to be lung adenocarcinoma after surgery. **B**: The lesion also has lobulation and spiculation signs, but the postoperative pathology is granuloma, this is not uncommon in clinical practice and is easily misdiagnosed as a malignant tumour by radiologists

On the basis of the inclusion and exclusion criteria, 420 patients with GNs and LADCs (LADCs, 281; GNs, 139; 231 males, 189 females; age range, 22–87 years; mean age, 55.93 ± 12.44 years). Samples of malignant and benign pulmonary nodules in this study are shown in Fig. 5. Cases from institution 1 were randomly divided into a TS and internal validation set IVS in a ratio of 7:3, while cases from institutions 2 and 3 were treated as the EVS. General clinical data of all patients are shown in Supplementary Table 1. Both the TS and IVS consisted of cases from institution 1 (*n* = 307), while cases of institutions 2 and 3 constituted the EVS (*n* = 113). The general clinical data of all three sets are shown in Supplementary Table 2.

### Backbone network selection and performance of networks without self-supervised pretext task training

**Fig. 6:**
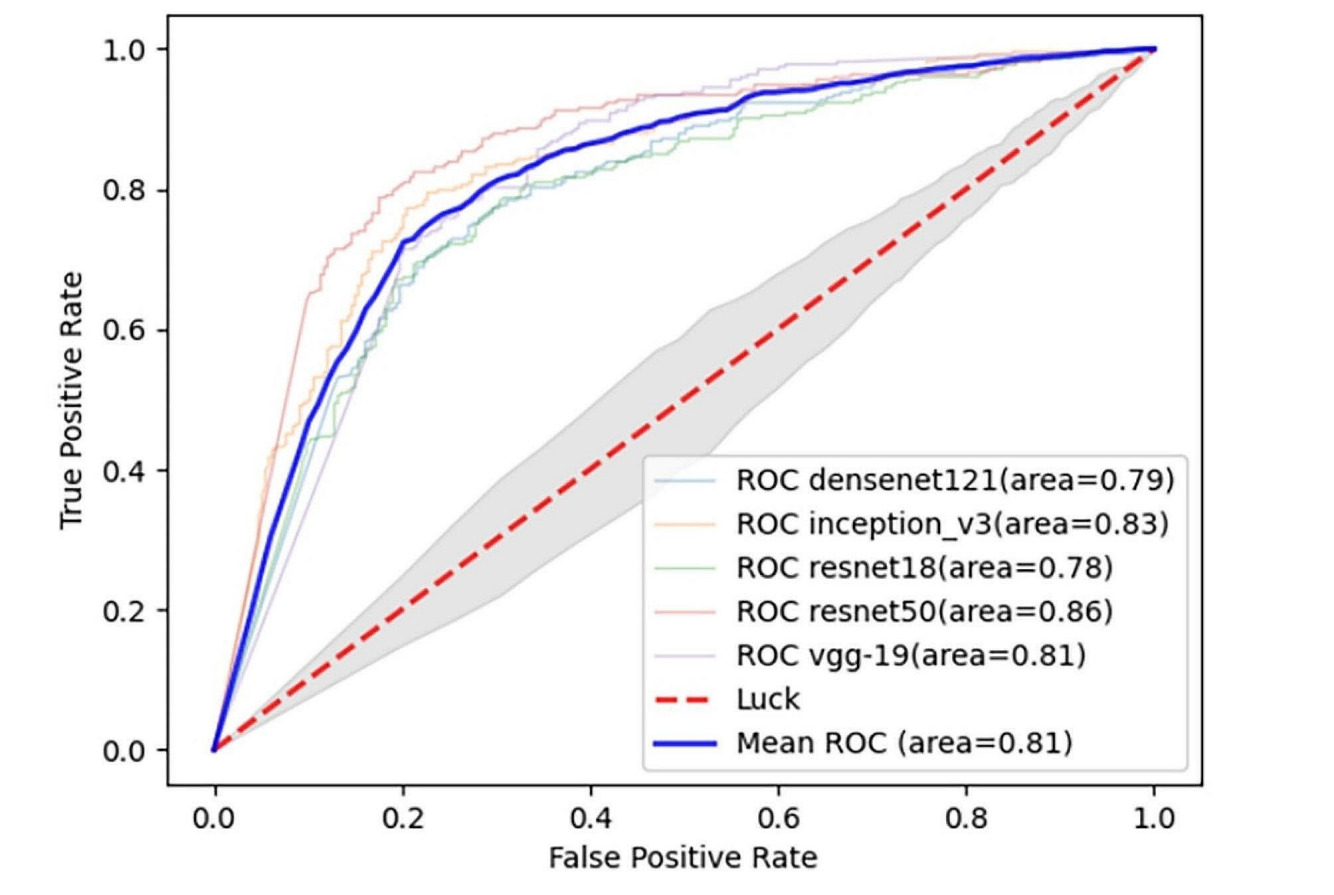
The ROC curves of the deep learning models built by different networks in the validation set

It has been reported that ResNet50 [17], DenseNet121 [18], Inception-v3 [19], ResNet18 [20] and VGG19 [21] networks are often used for classification tasks in the field of medical images and have achieved good performance. In order to select a more appropriate network, we pretrain with the above-mentioned network and parameters applied by previous researchers. The plain CT scan dataset (unrotated data) from Institution 1 was used for training and validation. The results show that the deep learning models built by DenseNet121, Inception-v3, ResNet18, ResNet50 and VGG19 networks have AUCs of 0.79, 0.83, 0.78, 0.86 and 0.81 in the validation set, with the best prediction performance for the ResNet50 network (AUC = 0.86). Therefore, we finally chose ResNet50 as the backbone network. The ROC curves of the deep learning models built by different networks in the validation set are shown in Fig. 6.

### Deep learning model and radiologist prediction performance

For the DL models based on NECT, VECT, and NEVECT images, the AUCs in the IVS and EVS were, respectively, 0.917, 0.876, and 0.896, and 0.889, 0.879, and 0.881. In contrast, the AUCs of assessments performed by radiologists 1, 2, 3, and 4 in the IVS and EVS were, respectively, 0.739, 0.783, 0.883, and 0.772 and 0.760, 0.760, 0.841 and 0.844. In the IVS and EVS, AUCs the of radiologist concordant results were 0.772 and 0.785, respectively. In addition, the comparison revealed that the performance of the rotated pre-trained network was higher than the non-rotated one (AUC = 0.86). The AUC, 95% CI, sensitivity, and specificity of the DL models and radiologists are shown in Table 1. The receiver operating characteristic curves of the DL models are shown in Supplementary Fig. 2, and those of the radiologists are shown in Supplementary Fig. 3.

### Radiologist diagnostic results and consistency test

Junior radiologists generally showed higher diagnostic accuracy for identifying solid LADCs than GNs, with a significant difference in the IVS (*P* < 0.001). However, the senior radiologists showed no definite patterns for the diagnostic accuracy of both types of lesions. The radiologists’ diagnostic results are shown in Supplementary Table 3. The diagnostic results of radiologists were tested for consistency. The diagnostic consistency among senior radiologists is high (highest kappa value, 0.801), the consistency among junior radiologists is medium (0.4 < kappa < 0.75), and the consistency between senior radiologists and junior radiologists is low (lowest kappa value, 0.282).The results of radiologists’ diagnosis consistency test are shown in Supplementary Table 4.

### Comparison of prediction performance

Delong test showed a significant difference in the prediction performance between the non-enhanced and venous contrast-enhanced CT DL models in the IVS (*p* = 0.001), with non-enhanced CT showing better prediction performance. No other significant differences were observed in the prediction performance of the DL models (all *P* > 0.05). Delong test also showed that the prediction performance of radiologists with the same experience level was not significantly different (all *P* > 0.05); the prediction performance of senior radiologists was higher than that of junior radiologists, and the difference was statistically significant in the IVS. The Delong test results of the predictive performance of the DL models and radiologists are presented in Supplementary Tables 5 and 6, respectively.

## Discussion

Lung adenocarcinomas require early resection while granulomatous nodules do not, indicating the importance of preoperative identification of these lesions. The biological information of tumors can be characterized by specific CT signs [22], with lobulation [23] and spiculation [24] both often associated with malignant tumors. However, solitary pulmonary nodules showing these signs have been pathologically proven to be granulomatous nodules [5, 6]. Therefore, we used self-supervised transfer learning and the ResNet50 network to establish a deep learning model for distinguishing granulomatous nodules and solid lung adenocarcinomas. The model showed maximum area under the curve values of 0.917 and 0.889 in the internal and external validation sets, respectively, highlighting the usefulness of this model in distinguishing these lesions and thereby facilitating preoperative diagnosis.

Self-supervised learning is an unsupervised learning method in which the model uses information from the data to maximize its knowledge reserves [25]. The accuracy of DL is directly proportional to the number of network layers within a reasonable range, but when the depth exceeds a certain threshold, gradient explosion and gradient dissipation problems can reduce the accuracy of the training set [22]. Resnet50 can improve the system performance of the network while increasing the depth [26, 27]. Therefore, this study used self-supervised transfer learning and the ResNet50 network to establish a DL model.

Radiomics is susceptible to CT acquisition data, lesion segmentation, feature extraction and modelling methods. Unlike radiomics, DL extracts features through end-to-end deep convolutional neural networks, and as a data-driven algorithm, deep learning-based models can achieve higher performance by constructing large datasets. [28, 29]. A radiomics model for distinguishing GNs with lobulation and spiculation signs from solid LADCs has been previously reported [30], in which the AUCs of non-enhanced, venous contrast-enhanced, and non-enhanced with venous contrast-enhanced CT models in the validation set were 0.817, 0.837, and 0.841. However, the DL model in this study showed better performance for predicting GNs and LADCs, indicating that DL models offer more advantages than radiomics models for distinguishing GNs with lobulation and spiculation signs and solid LADCs before operation.

Many recent studies have also used DL for prediction of benign and malignant pulmonary nodules. Yang et al. [31] established a DL model to predict benign and malignant lung nodules (AUC = 0.84), while Feng et al. [32] performed a retrospective analysis of tuberculous GNs and LADCs and obtained AUCs of 0.889, 0.879, and 0.809, respectively, in the test set, IVS and EVS, respectively. This study focused on distinguishing GNs and solid LADCs, and the maximum AUCs in the IVS and EVS were 0.917 and 0.889, respectively, which are higher than the results obtained by Feng et al. [32]. Thus, a DL model based on self-supervised transfer learning and Resnet50 shows great potential for distinguishing between GNs with lobulation and spiculation signs from solid LADCs and thereby facilitating preoperative diagnosis.

Our study also showed that diagnostic consistency was high, medium, and low among senior radiologists, among junior radiologists, and between senior and junior radiologists, highlighting inconsistencies in radiologist assessments. The lack of effective and unified diagnostic standards for distinguishing between these lesions and the presence of lobulation and spiculation signs can impair junior radiologists’ judgment. Moreover, traditional image diagnosis is based on evaluating the morphological characteristics of the focus and prior knowledge, resulting in differences in the diagnoses performed by different radiologists. The prediction performance of senior radiologists was higher than that of junior radiologists, further highlighting the influence of diagnostic experience on radiologists’ ability to distinguish between GNs with lobulation and spiculation signs and solid LADCs.

The AUCs of the DL model in this study were higher than the AUCs for radiologists, suggesting that the DL model shows obvious advantages over radiologist assessments in differentiating GNs and LADCs. In addition, the specificity of the DL was generally higher than that of the radiologists’, however, its diagnostic sensitivity was generally lower than that of the radiologists’. This may be related to the fact that the lung nodules included in this study had lobulation and spiculation signs. Lobulation and spiculation signs are usually indicative of malignancy, and radiologists are more likely to diagnose lung adenocarcinoma when these two signs are present in a lung nodule, influenced by subjective a priori knowledge. Therefore, radiologists have higher diagnostic sensitivity but lower specificity. Deep learning models, on the other hand, are completely data-driven and unaffected by subjective a priori knowledge, and thus have an advantage in diagnostic specificity.

In this study, the performance of the DL models with unenhanced CT was higher than that with CT enhanced scans, indicating that unenhanced CT may offer advantages over CT enhanced scans when using DL models. This may have occurred because the contrast agent residues in the tissue gaps of the focus on enhanced scans will interfere with the model’s evaluation of the internal structure of the focus, resulting in degradation of its prediction performance.Therefore, in clinical practice, DL models with unenhanced CT are more applicable and cost-effective in the screening and follow-up of pulmonary nodules.

## Limitations

Our study had some limitations. First, this was a retrospective study, which may have led to selection bias. Second, this study only compared GNs with LADCs, and did not include other inflammatory nodules, squamous cell carcinomas, and other tumors. Third, due to incomplete clinical data for parameters such as smoking history and tumor markers, this study did not analyze the influence of clinical risk factors on the predictive performance of the DL model.

## Conclusion

In conclusion, a CT deep learning model could effectively distinguishing granulomatous nodules with lobulation and spiculation signs from solid lung adenocarcinomas, and its diagnostic performance and specificity are superior to those of radiologists. Therefore, in clinical practice, when radiologists encounter SPNs with lobulation and spiculation signs that are difficult to characterise, CT deep learning, as a noninvasive and highly repeatable approach, can help to assist radiologists with differential diagnosis in the preoperative period and provide a theoretical basis for development of appropriate clinical diagnosis and treatment plans. In addition, our study also found that the non-enhanced model performs better than the enhanced one. Therefore, this method can also be used in patients with contraindications to enhancement, such as contrast allergies.

**Table 1:** The AUC, 95% CI, sensitivity, and specificity of the deep learning models and radiologists

	IVS					EVS			
	AUC	95%CI	SEN	SPE		AUC	95%CI	SEN	SPE
NECT	0.917	0.877–0.946	0.894	0.805		0.889	0.850–0.920	0.786	0.853
VECT	0.876	0.831–0.912	0.852	0.770		0.879	0.839–0.912	0.841	0.830
NEVECT	0.896	0.867–0.920	0.783	0.851		0.881	0.854–0.905	0.791	0.853
Radiologist1	0.739	0.686–0.787	0.905	0.573		0.760	0.670–0.835	0.729	0.791
Radiologist2	0.783	0.733–0.828	0.910	0.656		0.760	0.671–0.836	0.800	0.721
Radiologist3	0.883	0.841–0.916	0.848	0.917		0.841	0.760–0.903	0.729	0.953
Radiologist4	0.901	0.863–0.932	0.938	0.865		0.844	0.763–0.905	0.943	0.744
RCR	0.772	0.721–0.818	0.911	0.625		0.785	0.679–0.856	0.871	0.698

*Abbreviation* AUC: area under the curve, CI: confidence interval, IVS: internal validation set, EVS: external validation set, SEN: sensitivity, SPE: specificity, NECT: non-enhanced CT, VECT: venous contrast-enhanced CT, NEVECT: non-enhanced with venous contrast-enhanced CT, RCR: Radiologist concordant results

### Electronic supplementary material

Below is the link to the electronic supplementary material.

Supplementary Material 1

Supplementary Material 2

## Data Availability

No datasets were generated or analysed during the current study.

## Abbreviations

DL: Deep learning
GNs: Granulomatous nodules
LADCs: Lung adenocarcinomas
NECT: Non-enhanced CT
VECT: Contrast-enhanced CT
TS: Training set
IVS: Internal validation set
EVS: External validation se
AUC: Area under the curve
NEVECT: Non-enhanced with venous contrast-enhanced CT
SPNs: Solitary pulmonary nodules

## References

[1] Bueno J, Landeras L, Chung JH. Updated Fleischner Society Guidelines for managing Incidental Pulmonary nodules: common questions and challenging scenarios. Radiographics. 2018;38(5):1337–50. 30207935 10.1148/rg.2018180017

[2] Brandman S, Ko JP. Pulmonary nodule detection, characteri-zation, and management with multidetector computed tomography. J Thorac Imaging. 2011;26(2):90–105. 21508732 10.1097/RTI.0b013e31821639a9

[3] Aberle DR, Adams AM, Berg CD, et al. Reduced lung-cancer mortality with low-dose computed tomographic screening. N Engl J Med. 2011;365(5):395–409. 21714641 10.1056/NEJMoa1102873 PMC4356534

[4] Suzuki K, Li F, Aoyama M, et al. Effect of CAD on radiologists’ responses in distinction between malignant and benign pulmonary nodules on high-resolution CT. Geochim Cosmochim Acta. 2017;204(4):52–67.

[5] Patel VK, Naik SK, Naidich DP, et al. A practical algorithmic approach to the diagnosis and management of solitary pulmonary nodules: part 1: radiologic characteristics and imaging modalities. Chest. 2013;143(3):825–39. 23460160 10.1378/chest.12-0960

[6] Patel VK, Naik SK, Naidich DP, et al. A practical algorithmic approach to the diagnosis and management of solitary pulmonary nodules: part 2: pretest probability and algorithm. Chest. 2013;143(3):840–6. 23460161 10.1378/chest.12-1487

[7] Groheux D, Quere G, Blanc E, et al. FDG PET-CT for solitary pulmonary nodule and lung cancer: literature review. Diagn Interv Imaging. 2016;97(10):1003–17. 27567555 10.1016/j.diii.2016.06.020

[8] Gould MK, Maclean CC, Kuschner WG, et al. Accuracy of positron emission tomography for diagnosis of pulmonary nodules and mass lesions: a meta-analysis. JAMA. 2001;285(7):914–24. 11180735 10.1001/jama.285.7.914

[9] Koo CW, Lu A, Takahashi EA, et al. Can MRI contribute to pulmonary nodule analysis? J Magn Reson Imaging. 2019;49(7):e256–64. 30575193 10.1002/jmri.26587

[10] Yuan M, Zhang YD, Zhu C, et al. Comparison of intravoxel incoherent motion diffusion- weighted MR imaging with dynamic contrast-enhanced MRI for differentiating lung cancer from benign solitary pulmonary lesions. J Magn Reson Imaging. 2016;43(3):669–79. 26340144 10.1002/jmri.25018

[11] Cheng JZ, Ni D, Chou YH, et al. Computer-aided diagnosis with Deep Learning Architecture: applications to breast lesions in US images and pulmonary nodules in CT scans. Sci Rep. 2016;6:24454. 27079888 10.1038/srep24454 PMC4832199

[12] Avanzo M, Stancanello J, Pirrone G, et al. Radiomics and deep learning in lung cancer. Strahlenther Onkol. 2020;196(10):879–87. 32367456 10.1007/s00066-020-01625-9

[13] Chan HP, Samala RK, Hadjiiski LM, et al. Deep learning in Medical Image Analysis. Adv Exp Med Biol. 2020;1213:3–21. 32030660 10.1007/978-3-030-33128-3_1 PMC7442218

[14] Yoon H, Lee KY, Bechwati I. CLIMAR: classified linear interpolation based metal artifact reduction for severe metal artifact reduction in x-ray CT imaging. Phys Med Biol. 2021; 66(7). 10.1088/1361-6560/abeae6 33647890

[15] Jing LL, Tian YL. Self-supervised visual feature learning with deep neural networks: a Survey. IEEE Trans Pattern Anal Mach Intell. 2020;PP(99):1–1. 10.1109/TPAMI.2020.2992393 32386141

[16] Ntelemis F, Jin Y, Thomas SA. A generic self-supervised Framework of LearningInvariant discriminative features. IEEE Trans Neural Netw Learn Syst. 2023;PP. 10.1109/TNNLS.2023.3265607 37126634

[17] Islam W, Jones M, Faiz R, et al. Improving performance of breast lesion classification using a ResNet50 model optimized with a novel attention mechanism. Tomography. 2022;8(5):2411–25. 36287799 10.3390/tomography8050200 PMC9611554

[18] Tharmaseelan H, Vellala AK, Hertel A, et al. Tumor classification of gastrointestinal liver metastases using CT-based radiomics and deep learning. Cancer Imaging. 2023;23(1):95. 37798797 10.1186/s40644-023-00612-4 PMC10557291

[19] Coudray N, Ocampo PS, Sakellaropoulos T, et al. Classification and mutation prediction from non-small cell lung cancer histopathology images using deep learning. Nat Med. 2018;24(10):1559–67. 30224757 10.1038/s41591-018-0177-5 PMC9847512

[20] Liu Y, She GR, Chen SX. Magnetic resonance image diagnosis of femoral head necrosis based on ResNet18 network. Comput Methods Programs Biomed. 2021;208:106254. 34260970 10.1016/j.cmpb.2021.106254

[21] Khan MA, Rajinikanth V, Satapathy SC, et al. VGG19 Network assisted Joint Segmentation and classification of lung nodules in CT images. Diagnostics (Basel). 2021;11(12):2208. 34943443 10.3390/diagnostics11122208 PMC8699868

[22] Jain D, Roy-Chowdhuri S. Molecular Pathology of Lung Cancer cytology specimens: a concise review. Arch Pathol Lab Med. 2018;142(9):1127–33. 29547001 10.5858/arpa.2017-0444-RA

[23] Cruickshank A, Stieler G, Ameer F. Evaluation of the solitary pulmonary nodule. Intern Med J. 2019;49(3):306–15. 30897667 10.1111/imj.14219

[24] Ko JP, Bagga B, Gozansky E, et al. Solitary pulmonary nodule evaluation:pearls and pitfalls. Semin Ultrasound CT MR. 2022;43(3):230–45. 35688534 10.1053/j.sult.2022.01.006

[25] Nadif M. F Role 2021 Unsupervised and self-supervised deep learning approaches for biomedical text mining. Brief Bioinform 22 2 1592–603. 33569575 10.1093/bib/bbab016

[26] He K, Zhang X, Ren S et al. Deep residual learning for image recognition. 2016 IEEE Conference on Computer Vision and Pattern Recognition (CVPR). 2016; 770–778.

[27] Alaeddine H, Jihene M. Deep Residual Network in Network. Computational Intelligence and Neuroscience. 2021; 2021:6659083. 10.1155/2021/6659083 PMC7925065 33679966

[28] Kurland BF, Gerstner ER, Mountz JM, et al. Promise and pitfalls of quantitative imaging in oncology clinical trials. Magn Reson Imaging. 2012;30(9):1301–12. 22898682 10.1016/j.mri.2012.06.009 PMC3466405

[29] Parekh VS, Jacobs MA. Deep learning and radiomics in precision medicine. Expert Rev Precis Med Drug Dev. 2019;4(2):59–72. 31080889 10.1080/23808993.2019.1585805 PMC6508888

[30] Yang X, He J, Wang J, et al. CT-based radiomics signature for differentiating solitary granulomatous nodules from solid lung adenocarcinoma. Lung Cancer. 2018;125:109–14. 30429007 10.1016/j.lungcan.2018.09.013

[31] Yang K, Liu J, Tang W, et al. Identification of benign and malignant pulmonary nodules on chest CT using improved 3D U-Net deep learning framework. Eur J Radiol. 2020;129:109013. 32505895 10.1016/j.ejrad.2020.109013

[32] Feng B, Chen X, Chen Y, et al. Solitary solid pulmonary nodules: a CT-based deep learning nomogram helps differentiate tuberculosis granulomas from lung adenocarcinomas. Eur Radiol. 2020;30(12):6497–507. 32594210 10.1007/s00330-020-07024-z

