# Supplementary material for: Differentiation of granulomatous nodules with lobulation and spiculation signs from solid lung adenocarcinomas using a CT deep learning model
Source: BMC Cancer. 2024 Jul 22;24:875. doi: 10.1186/s12885-024-12611-0 (PMC11265160; doi:10.1186/s12885-024-12611-0)
Supplement: Supplementary file 1 — Supplementary Material 1 [file 12885_2024_12611_MOESM1_ESM.docx]

**Supplementary Figure**


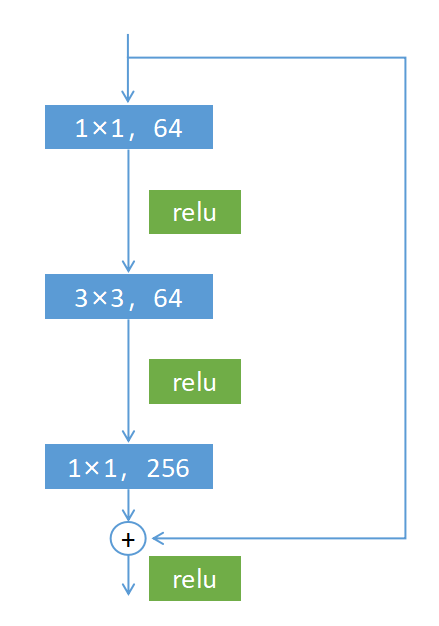


**Supplementary figure 1.** The residual unit’s structure. Each residual unit is composed of multiple cascaded convolution layers and a cross-layer right angle edge. Relu refers to the activation function, which can accept the signal output from the previous unit and convert it into a form that can be received by the next unit.


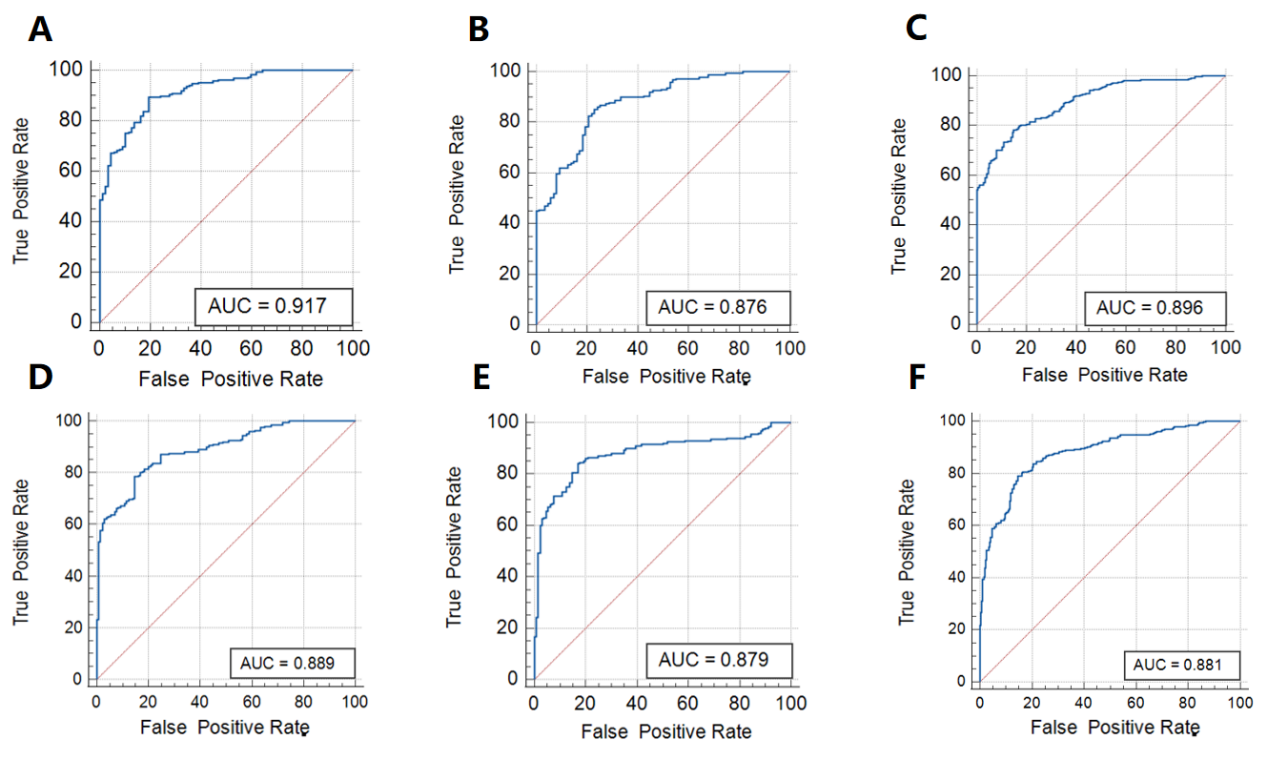


**Supplementary figure 2.** The receiver operating characteristic curves of deep learning models. (A), (B) and (C) show the curves of the models based on non-enhanced, venous contrast-enhanced, and non-enhanced with venous contrast-enhanced CT in the internal validation set, respectively, while (D), (E), and (F) show the corresponding curves in the external validation set.


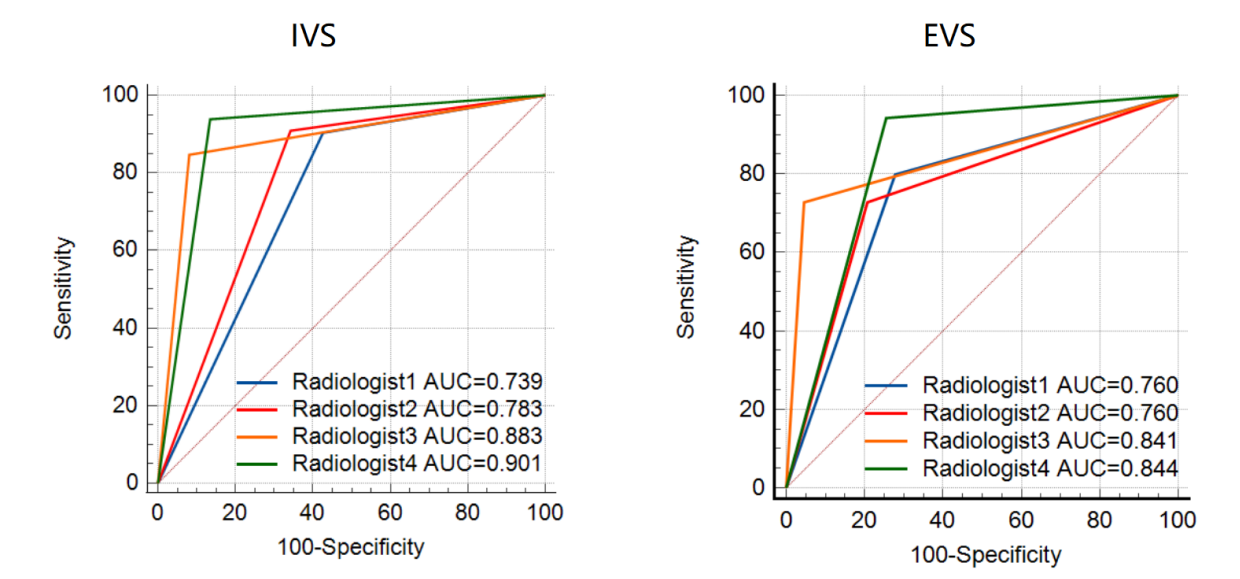


**Supplementary figure 3.** The receiver operating characteristic curves for radiologist assessments. IVS = internal validation set, EVS = external validation set.
