# Supplementary material for: Differentiation of granulomatous nodules with lobulation and spiculation signs from solid lung adenocarcinomas using a CT deep learning model
Source: BMC Cancer. 2024 Jul 22;24:875. doi: 10.1186/s12885-024-12611-0 (PMC11265160; doi:10.1186/s12885-024-12611-0)
Supplement: Supplementary file 2 — Supplementary Material 2 [file 12885_2024_12611_MOESM2_ESM.docx]

**Supplementary Table**

**Supplementary table 1**

General clinical data of all patients

|  |  | LADC (n = 281) | GN (n = 139) | Statistic | P |
| --- | --- | --- | --- | --- | --- |
| Sex | M | 134 | 97 | 18.347 | <0.001 |
|  | F | 147 | 42 |  |  |
| [Average age](javascript:;) (y) | | 58.62 ± 11.75 | 50.49 ± 12.04 | -6.616 | <0.001 |
| Lesion diameter (cm) | | 2.28 ± 0.55 | 1.86 ± 0.65 | -6.542 | <0.001 |

Notes: LADC = Lung adenocarcinoma; GN = Granulomatous nodule.

**Supplementary table 2**

General clinical data of the training, internal validation, and external validation sets

|  |  | TS/IVS (n = 307) | EVS (n = 113) | Statistic | P |
| --- | --- | --- | --- | --- | --- |
| [Pathology](javascript:;) | LADC | 211 | 70 | 1.716 | 0.190 |
|  | GN | 96 | 43 |  |  |
| Sex | M | 174 | 57 | 4.388 | 0.036 |
|  | F | 133 | 56 |  |  |
| [Average age](javascript:;) (y) | | 55.96 ± 13.06 | 55.85 ± 10.63 | -0.087 | 0.931 |
| Diameter of lesion (cm) | | 2.14 ± 0.63 | 2.14 ± 0.59 | 0.110 | 0.913 |

Note: TS = Test set; IVS = Internal validation set; EVS = External validation set; LADC = Lung adenocarcinoma; GN = Granulomatous nodule.

**Supplementary table 3**

Diagnostic results obtained by radiologists

|  | Radiologist diagnosis | | [Pathological diagnosis](javascript:;) | | Statistic | P |
| --- | --- | --- | --- | --- | --- | --- |
|  |  |  | LADC | GN |  |  |
| IVS (n = 307) | Radiologist1 | LADC | 191（0.905） | 41（0.427） | 45.759 | <0.001 |
|  |  | GN | 20（0.095） | 55（0.573） |  |  |
|  | Radiologist2 | LADC | 192（0.910） | 33（0.344） | 30.186 | <0.001 |
|  |  | GN | 19（0.090） | 63（0.656） |  |  |
|  | Radiologist3 | LADC | 179（0.848） | 8（0.083） | 2.718 | 0.99 |
|  |  | GN | 32（0.151） | 88（0.917） |  |  |
|  | Radiologist4 | LADC | 198（0.938） | 13（0.135） | 4.636 | 0.031 |
|  |  | GN | 13（0.062） | 83（0.865） |  |  |
| EVS (n = 113) | Radiologist1 | LADC | 51（0.729） | 9（0.209） | 0.552 | 0.458 |
|  |  | GN | 19（0.271） | 34（0.791） |  |  |
|  | Radiologist2 | LADC | 56（0.800） | 12（0.279） | 0.940 | 0.332 |
|  |  | GN | 14（0.200） | 31（0.721） |  |  |
|  | Radiologist3 | LADC | 51（0.729） | 2（0.047） | 8.906 | 0.003 |
|  |  | GN | 19（0.271） | 41（0.953） |  |  |
|  | Radiologist4 | LADC | 66（0.943） | 11（0.256） | 9.133 | 0.003 |
|  |  | GN | 4（0.057） | 32（0.744） |  |  |

Note: LADC = Lung adenocarcinoma; GN = Granulomatous nodule; IVS = Internal validation set; EVS = External validation set.

**Supplementary table 4**

Consistency results for radiologists' diagnosis

|  | IVS | | | |  | EVS | | | |
| --- | --- | --- | --- | --- | --- | --- | --- | --- | --- |
|  | R 1 | R 2 | R 3 | R 4 |  | R 1 | R 2 | R 3 | R 4 |
| R 1 | / | 0.495 | 0.421 | 0.492 |  | / | 0.534 | 0.282 | 0.282 |
| R2 | 0.495 | / | 0.565 | 0.574 |  | 0.534 | / | 0.524 | 0.497 |
| R3 | 0.421 | 0.565 | / | 0.801 |  | 0.282 | 0.524 | / | 0.634 |
| R4 | 0.492 | 0.574 | 0.801 | / |  | 0.282 | 0.497 | 0.634 | / |

Note: IVS =Internal validation set; EVS = External validation set; R = Radiologist.

**Supplementary table 5**

Delong test results of the predictive performance of the deep learning models

|  | IVS | | |  | EVS | | |
| --- | --- | --- | --- | --- | --- | --- | --- |
|  | NECT | VECT | NEVECT |  | NECT | VECT | NEVECT |
| NECT | / | 0.001 | 0.051 |  | / | 0.351 | 1.000 |
| VECT | 0.001 | / | 0.714 |  | 0.351 | / | 0.951 |
| NEVECT | 0.051 | 0.714 | / |  | 1.000 | 0.951 | / |

Note: IVS = Internal validation set; EVS = External validation set; NECT = Non-enhanced CT; VECT = Venous enhanced CT; NEVECT = non-enhanced with venous enhanced CT.

**Supplementary table 6**

Delong test results for radiologists’ predictive performance

|  | IVS | | | |  | EVS | | | |
| --- | --- | --- | --- | --- | --- | --- | --- | --- | --- |
|  | R 1 | R 2 | R 3 | R 4 |  | R 1 | R 2 | R 3 | R 4 |
| R 1 | / | 0.157 | <0.001 | <0.001 |  | / | 0.986 | 0.093 | 0.100 |
| R2 | 0.157 | / | <0.001 | <0.001 |  | 0.986 | / | 0.058 | 0.084 |
| R3 | <0.001 | <0.001 | / | 0.301 |  | 0.093 | 0.058 | / | 0.954 |
| R4 | <0.001 | <0.001 | 0.301 | / |  | 0.100 | 0.084 | 0.954 | / |

Note: IVS = Internal validation set; EVS = External validation set; R = Radiologist.
